# Anaplastic Lymphoma Kinase (ALK)-Negative Anaplastic Large Cell Non-Hodgkin Lymphoma as a Rare Differential Diagnosis of Lung Cancer: A Case Report

**DOI:** 10.7759/cureus.55258

**Published:** 2024-02-29

**Authors:** Alondra Esthefanía Llamas Domínguez, Julio A Palma Zapata, Silvia Denise Ponce Campos, Juliana Palma Zapata, Elvia Jacobo Medrano, Pedro Cisneros Garza

**Affiliations:** 1 Medicine, Autonomous University of Aguascalientes, Aguascalientes, MEX; 2 Pneumology, Institute of Security and Social Services for State Workers, Aguascalientes, MEX; 3 Hematology, Institute of Security and Social Services for State Workers, Aguascalientes, MEX; 4 Internal Medicine, Institute of Security and Social Services for State Workers, Aguascalientes, MEX

**Keywords:** anaplastic lymphoma kinase (alk), cd30, peripheral t-cell lymphoma, mature t-cell lymphoma, poorly differentiated lung adenocarcinoma, anaplastic large cell lymphoma (alcl)

## Abstract

Anaplastic large cell lymphomas (ALCL) are a group of sporadic malignancies that generally have an aggressive clinical course, especially the subtype of anaplastic lymphoma kinase (ALK)-negative ALCL. The appropriate diagnostic study modalities must be chosen to make an accurate diagnosis and promptly initiate specific treatment. We present the clinical case of a 72-year-old male patient with dyspnea on small efforts accompanied by diaphoresis and a weight loss of 10 kg in two months. Physical examination revealed adenopathy in the cervical region and bilateral pleural effusion. The pleural and lung biopsies revealed poorly differentiated metastatic adenocarcinomas. A multidisciplinary analysis was carried out; the typical clinical-radiographic presentation of adenocarcinoma was ruled out with immunohistochemistry, thus determining a diagnosis of ALK-negative anaplastic large cell non-Hodgkin's lymphoma. This case represented a diagnostic and therapeutic challenge since it is a rare entity with a poor prognosis, and there are only a few studies about the choice of appropriate chemotherapy in these patients.

## Introduction

Non-Hodgkin lymphomas (NHL) are a diverse group of lymphoid malignancies. About 85% to 90% of NHL arise from B cells, and 10% to 15% originate from T cells or NK cells. It is the most common type of lymphoma affecting the mediastinum. Mediastinal NHL affects the mediastinal lymph nodes, thymus, and extranodal lesions such as the heart, lung, pleura, and pericardium [1]. According to the WHO classification of haematolymphoid tumors (5th edition), anaplastic large cell lymphoma (ALCL) is a rare type of mature T-cell NHL (accounting for approximately 2% of NHL cases). It is characterized by the intense and uniform expression of CD30. This group of malignant neoplasms is typically associated with an aggressive clinical course and a high frequency of extranodal lesions [2-5]. It is more prevalent in regions such as North America and Europe and shows a strong predilection for the male gender. Conversely, it is less common in South America and Asia [3]. Anaplastic large cell lymphoma is categorized into two subtypes: anaplastic lymphoma kinase (ALK)-positive ALCL and anaplastic lymphoma kinase (ALK)-negative ALCL [3].

The ALK-negative ALCL has a worse prognosis and is usually diagnosed in older patients, with a peak between the ages of 40 and 65. The symptoms of this condition include rapidly progressive lymphadenopathy and constitutional symptoms. The diagnosis is usually made at an advanced stage, with high levels of lactate dehydrogenase (LDH) and an intermediate-high or high international prognostic index (IPI) score [6].

We present the case of a patient who was initially diagnosed with poorly differentiated lung adenocarcinoma. However, after a multidisciplinary clinical and immunohistopathological analysis, the correct diagnosis was concluded to be ALK-negative ALCL NHL. Even though this type of lymphoma has a low incidence, it is even rarer to involve the lungs. The lung variant of ALCL usually presents with mediastinal lymphadenopathy. It is essential to emphasize the significance of clinical and imaging correlation in patients with cancer, particularly those who do not display symptoms consistent with their anatomopathological diagnosis. In such cases, further analysis should be conducted through complementary studies such as immunohistochemistry, especially if poorly differentiated tumors are detected, to rule out uncommon causes that may have a worse prognosis. This is particularly relevant in cases like the one presented here.

## Case presentation

A 72-year-old male patient, a retired teacher, had a medical history with a biomass exposure index of 180 hours per year, while his community bacillus exposure test results were negative. He denied any history of smoking or exposure to organic and inorganic dust. The patient had received three immunizations against SARS-CoV2 but had not been immunized against influenza viruses. Prior to the onset of his current illness, he self-reported being in good health.

The individual's current medical state commenced in September of 2022, with the manifestation of progressive dyspnea, eventually reaching Medical Research Council (MMRC) level 3 and a weight reduction of 10 kg and night sweats over two months. He denied coughing and expectoration. The patient went to a doctor in another unit and received ceftriaxone and budesonide/formoterol treatment without improvement. His dyspnea worsened and progressed to dyspnea on minor exertion, accompanied by diaphoresis. He sought medical attention at the emergency department on October 10, 2022. Upon admission, his vital signs were recorded as follows: blood pressure 107/55 mmHg, respiratory rate 20 breaths per minute, heart rate 88 beats per minute, temperature 36°C (96.8°F), and oxygen saturation by pulse oximetry at 92% with a fraction of inspired oxygen (FiO2) of 21%.

During the physical examination, the patient appeared conscious and oriented but had slight paleness in their mucous membranes and integuments. There were no signs of jugular engorgement in the neck, but lymphadenopathy was present in the right anterior cervical region. Upon observation, it was noted that there was a decrease in thoracic movements. The patient had reduced bilateral breath sounds, decreased fremitus, and dullness on percussion. There were thick rales in the bilateral interscapulovertebral region, and normodynamic heart sounds were detected. The abdomen was soft, depressible, and not painful on medium or deep palpation. The patient's extremities were integer, without edema, and had immediate capillary refill.

**Figure 1:**
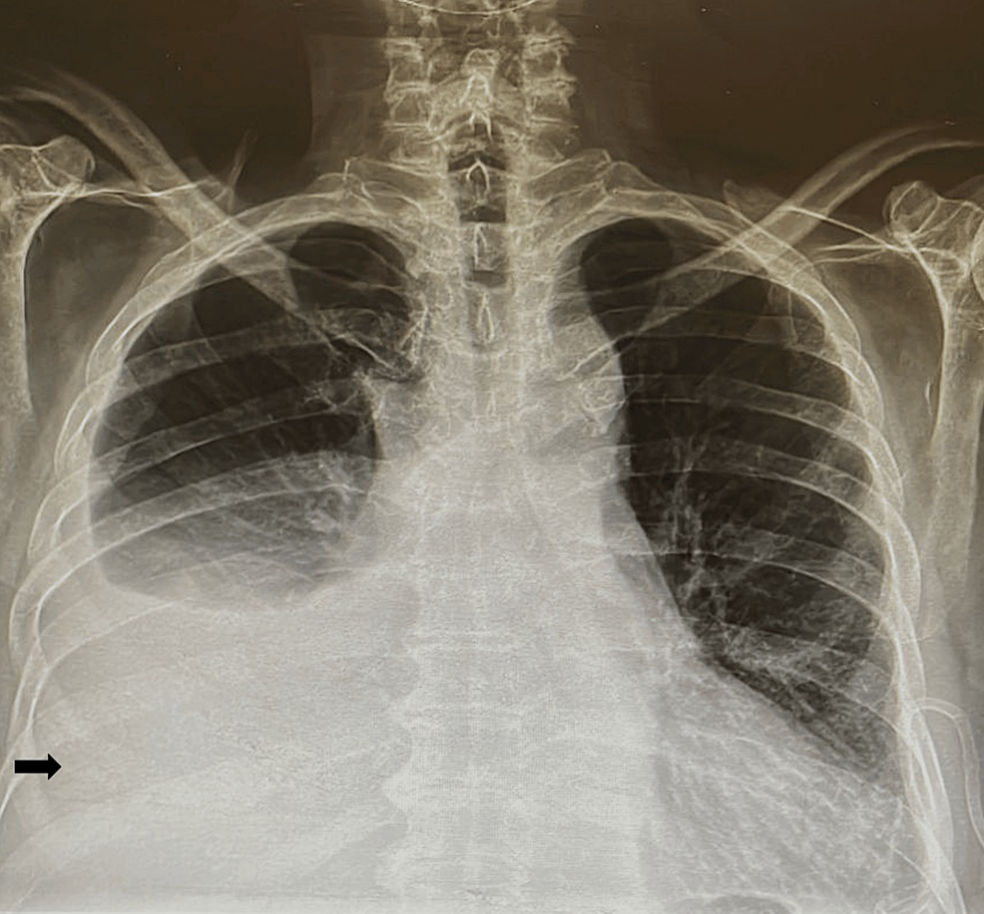
Chest X-ray at admission Upon admission, the chest x-ray revealed the presence of pleural effusion that was bilateral with a right-sided predominance (black arrow).

**Figure 2:**
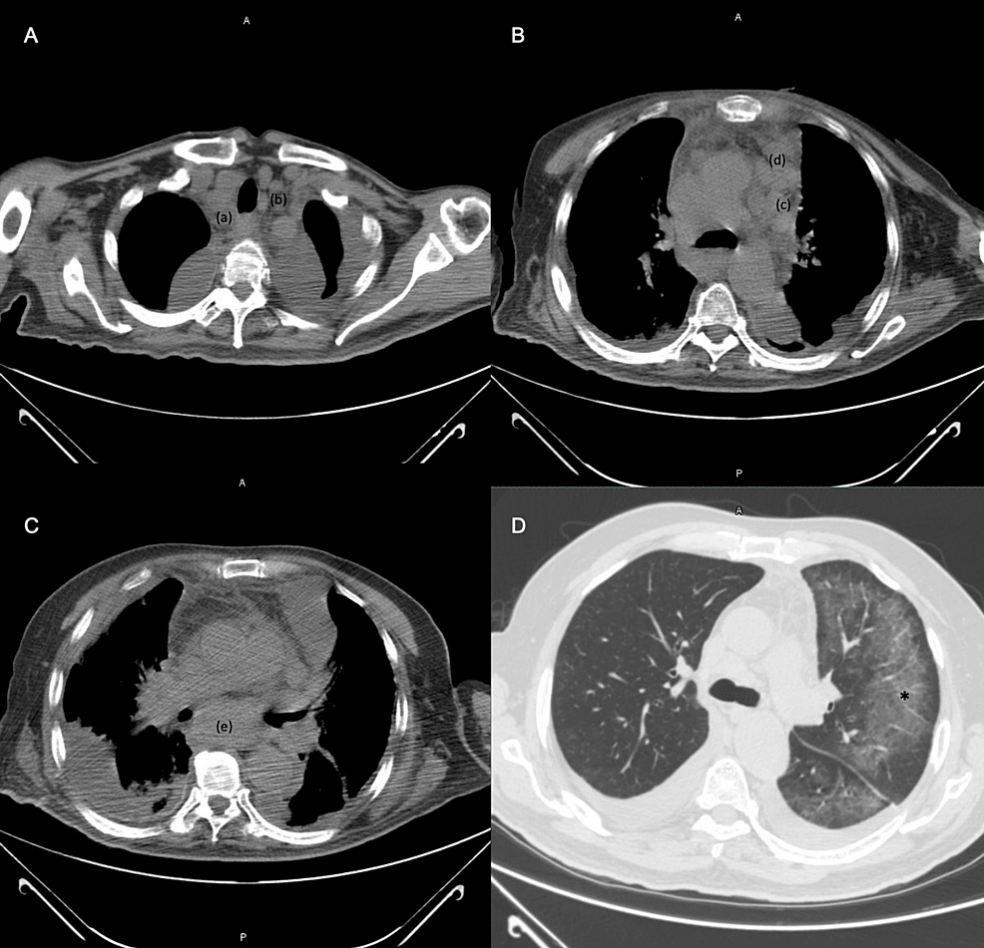
Simple chest CT conducted after thoracentesis with the mediastinal window setting A: The presence of lymphadenopathy is seen in regions 2R (a) and 2L (b), as well as bilateral pleural effusion; B: Lymph nodes in regions 5 (c) and 6 (d); C: Adenopathy in region 7 (e) and bilateral pleural effusion; D: Ground glass (*) seen in the left axial interstitium of the subpleural region

The presence of pleural effusion was confirmed by a chest radiograph (Figure 1) and CT, and it was decided to perform a bilateral thoracentesis. The pleural fluid analysis discovered that the patient had a complicated polymorphonuclear exudate (Table 1). Therefore, it was decided to begin him on an empirical antimicrobial treatment of meropenem 1 g every eight hours for seven days. This antibiotic treatment was selected after receiving the results of the patient's polymorphonuclear exudate test of pleural fluid. Additionally, the patient's condition had become increasingly complex by the fourth day of hospitalization. Different complementary studies were carried out within the diagnostic approach (Table 1, Figure 2).

**Table 1:** Results of laboratory studies at the time of admission *In this case, the estimated value of pleural fluid LDH is calculated to be 0.6 times the upper limit of serum LDH, which would be 168 IU/L. LDH: Lactate dehydrogenase

Laboratory studies	Test	Result	Normal value range
Blood test	Hemoglobin	15.7 g/dL	14-18 g/dL
	Mean corpuscular volume	92.5 fL	80-98 fL
	Platelet count	215,000/microL	150,000-450,000/microL
	Leukocyte count	8360/microL	4000-11,000/microL
	Glucose	113 mg/dL	70-99 mg/dL
	Urea nitrogen	17 mg/dL	8-20 mg/dL
	Creatinine	0.5 mg/dL	0.70-1.30 mg/dL
	LDH	368 U/L	140-280 U/L
	Brain natriuretic peptide	44.8 pg/mL	<100 pg/mL
Cytochemical examination of pleural fluid	Appearance	Cloudy	Clear
	Color	Yellow	Light yellow
	LDH	2861 UI/L	<0.6 times the upper limit of the laboratory's normal serum LDH*
	Proteins	4.9 g/dL	1-2 g/dL
	Glucose	20 mg/dL	Similar to serum
Cytological examination of pleural fluid	Leukocyte count	5670 cells/ mL	1716 cells/ mL
	Polymorphonuclear percentage	70%	<5%

The patient underwent a diagnostic workup that included a tumor marker evaluation and peripheral blood smear analysis. The results of these tests were within the normal range. A biopsy of the mediastinal adenopathy was subsequently performed using a bronchoscope and a 21G aspiration needle. The biopsy results were negative. 

**Figure 3:**
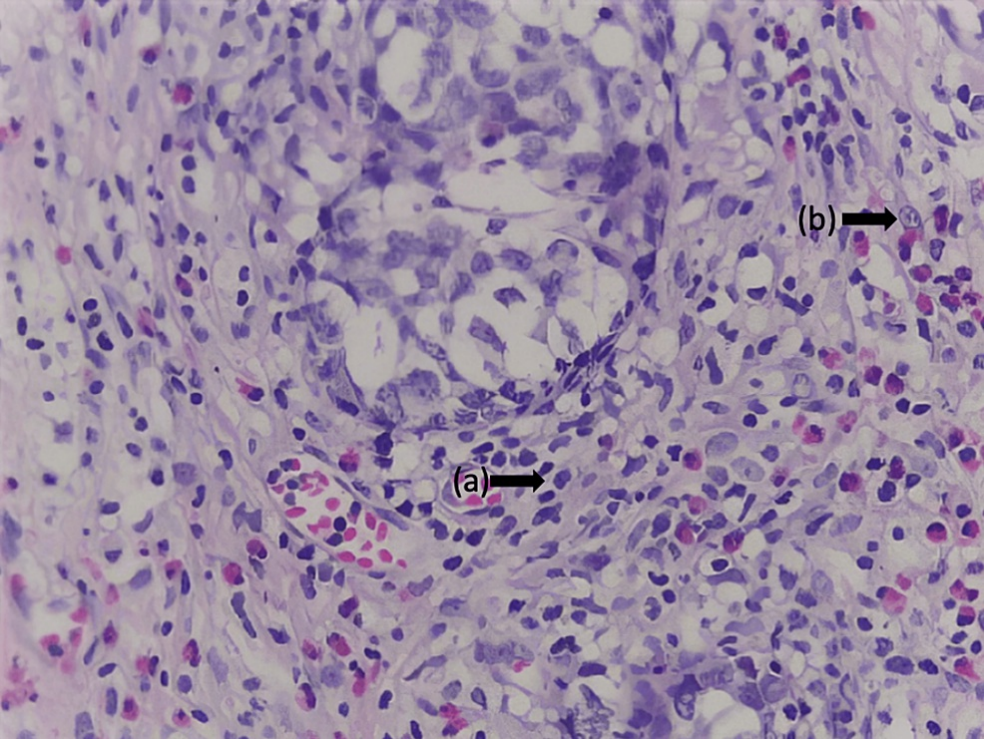
Lung parenchyma biopsy stained with H&E (40x) a: Neoplastic cells of epithelial origin of intermediate size, with moderate cytoplasm and large, hyperchromatic nuclei; b: Atypical mitotic figures These cells are arranged diffusely in nests and form poorly differentiated glandular structures. These neoplastic cells are also diffusely observed in nests and form glandular structures in the pleura and lymph nodes. H&E: Hematoxylin and eosin

Due to the inconclusive results of the previous study and the persisting suspicion of a possible malignant process, a bilateral thoracotomy was performed to conduct a pleural and lung biopsy. The biopsy results confirmed the presence of poorly differentiated metastatic adenocarcinoma (Figure 3), thus initiating treatment with carboplatin and pemetrexed; however, only one dose was administered.

**Figure 4:**
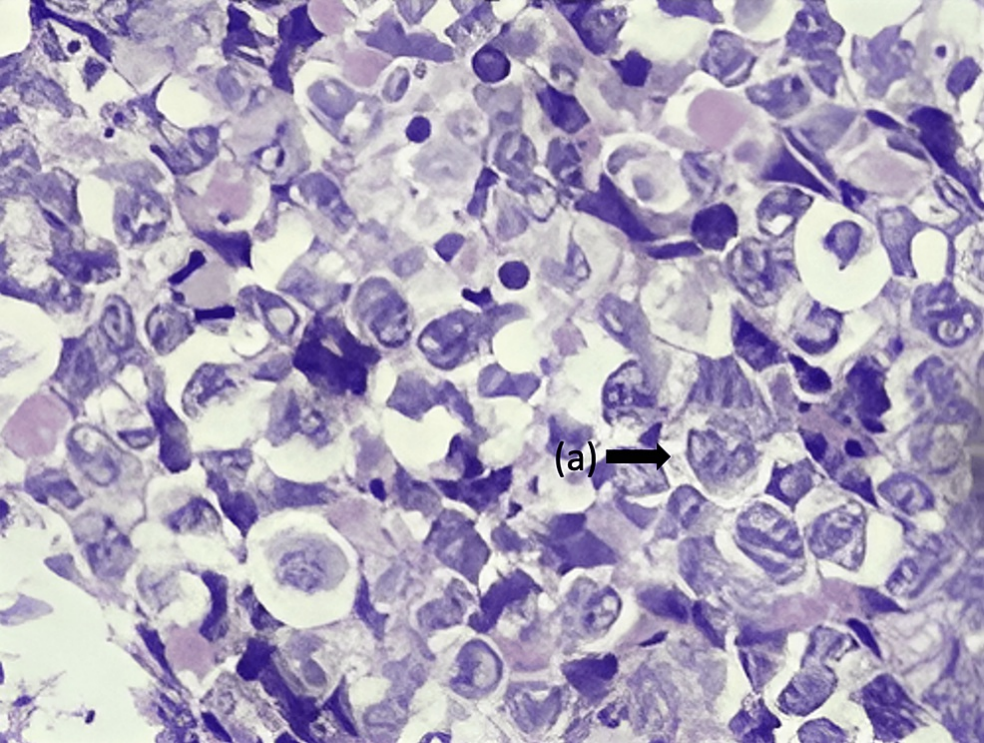
Lung parenchyma biopsy stained with H&E (400x) The histopathological examination showed a lymphoid proliferation consisting of large cells that exhibit cytoplasmic abundance featuring a centrally located nucleus of open chromatin that assumes a kidney-shaped configuration (a). These cells were observed to rest on a reactive background comprised of mature lymphocytes, abundant eosinophils, and macrophages. These features were also observed in the pleura and mediastinal nodes. H&E: Hematoxylin and eosin

**Figure 5:**
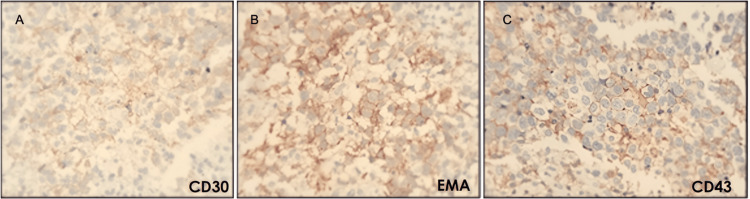
Positive immunostaining lung parenchyma biopsy indicating ALCL (40x) A: Positive staining for CD30; B: Positive staining for EMA, C: Positive staining for CD43. The presence of CD30 indicates the activation of the lymphoma cells, while EMA and CD43 are characteristic markers of ALCL. ALCL: Anaplastic large cell lymphomas, EMA: Epithelial membrane antigen

Despite the pathological diagnosis, the clinical and imaging findings did not conform to the typical presentation of adenocarcinoma, mainly due to the absence of a solid lung tumor and the negative report of tumor markers. Consequently, a multidisciplinary analysis was conducted, in which it was decided to repeat the histopathological study (Figure 4), and the case was reviewed using immunohistochemistry, which led to an updated diagnosis of ALK-negative ALCL, CD30 positive, epithelial membrane antigen (EMA) positive, leukosialin or sialophorin (CD43) positive, neural cell adhesion molecule (CD56) negative, and ALK-negative (Figure 5, Table 2). The patient received treatment with brentuximab vedotin every 21 days for 16 applications. Despite receiving medical attention and treatment, the patient ultimately passed away four months after being diagnosed.

**Table 2:** Immunohistochemistry report The immunohistochemistry report demonstrated a T cell profile related to ALCL characterized by CD30+, CD4+, CD45+, CD43+, and EMA antibodies positive. Notably, the report was negative for ALK. P63: Protein 63; TTF1: Thyroid transcription factor-1; ALK: Anaplastic lymphoma kinase; LMP1: Latent membrane proteins 1; EMA: Epithelial membrane antigen

Immunohistological markers	Result
P63	Negative
Chromogranin	Negative
TTF1	Negative
CKAE1/AE3	Negative
CD20	Negative
CD3	Negative
CD30	Positive
ALK	Negative
CD56	Negative
LMP1	Negative
EMA	Positive
CD15	Negative
CD4	Focal positive
PAX5	Negative
CD45	Focal positive
CD43	Positive

## Discussion

The present case posed a crucial challenge for clinicians in reaching the correct diagnosis due to the initial pathology report indicating a poorly differentiated metastatic adenocarcinoma. Therefore, the initial report's findings required critical evaluation to determine the course of action to ensure the patient's best possible outcome. Our patient's clinical presentation was marked by dyspnea, which is a common symptom in patients with malignant pleural effusion. However, up to a quarter of patients may not have trouble breathing. In addition to dyspnea, patients may also exhibit classic constitutional symptoms of oncological pathologies, such as fever, chills, fatigue, weakness, and weight loss [7].

Laboratory and imaging studies can provide valuable information in addition to clinical findings to aid in the initial evaluation. The initial laboratory results of our patient were within normal limits, except for increased LDH. Imaging confirmed the presence of pleural effusion (Figure 1) and showed the presence of adenopathies and ground glass opacity due to tumor activity from lymphoma (Figure 2). After performing thoracentesis and analyzing the characteristics of the pleural effusion, it was suspected to be a paraneoplastic effusion due to elevated protein concentration, pleocytosis, LDH greater than 1000 U/L, and a low glucose concentration in the pleural fluid (Table 1) [8].

Our case's fine needle aspirate did not produce satisfactory diagnostic results. This is a common issue with these types of aspirates, as they often need to provide sufficient tissue to determine the structural composition needed to diagnose all lymphoma subtypes with certainty. If the biopsy does not offer a definitive diagnosis, an additional study is based on the most probable diagnosis, considering the clinical, imaging, and pathological characteristics. Due to the high suspicion of malignancy, a thoracotomy with a biopsy was performed [7].

The diagnosis of well-differentiated adenocarcinomas is based on their papillary, glandular/acinar, or solid morphology and mucin production. The histology of these adenocarcinomas is characterized by the presence of cells with abundant cytoplasm, pleomorphic nuclei featuring a thick chromatin pattern, and glandular architecture. However, poorly differentiated carcinomas are challenging to classify into subcategories, such as adenocarcinoma, squamous cell carcinoma, or neuroendocrine neoplasms, due to a lack of classic morphological characteristics [9]. Additionally, extensive studies are required to diagnose poor differentiation or undifferentiation, such as an immunohistochemistry panel and mucin stains, to rule out differential diagnoses such as melanoma and lymphoma. Immunohistochemical staining is a useful diagnostic tool for adenocarcinomas, as over 70% of them express thyroid transcription factor-1 (TTF-1) and napsin A proteins, while cytokeratin 5/6 (CK5/6) or protein 63 (P63) are expressed less frequently [10].

Lung adenocarcinoma is a disease that shows a wide range of architectural and morphological profiles. The undifferentiated histological pattern of the biopsy (Figure 3), the presence of multiple lymphadenopathies without a solid lung tumor, and the negative tumor markers did not provide conclusive evidence for diagnosing adenocarcinoma. Therefore, a new biopsy was requested for immunostaining, which showed positive antibodies and compatible morphology for ALCL (Figures 4-5, Table 2).

The biopsy results showed the classic morphology of ALCL, with diffuse growth of large neoplastic cells, the characteristic shape of a kidney or horseshoe, and abundant cytoplasm (Figure 4). The immunohistochemical pattern showed that the cells were positive for CD30 but negative for ALK, which is consistent with the diagnosis of ALK-negative ALCL (Figure 5). The histopathological diagnosis requires a pathologist familiar with this type of neoplasm because its morphology can imitate poorly differentiated carcinoma, large B-cell lymphoma, and a syncytial variant of classical Hodgkin lymphoma, as some examples [2,3].

Anaplastic large cell lymphomas have been defined as aggressive systemic lymphomas, which are divided into two categories: ALK-positive ALCL and ALK-negative ALCL. Approximately 50% of ALCL cases are ALK-positive, wherein ALK fusion proteins are expressed. These proteins arise from the chromosomal rearrangement of the ALK gene, located on the short arm of chromosome 2 (2p23). The ALK gene can activate cell signaling pathways, leading to abnormal cell proliferation and growth, which ultimately results in malignancy. The morphological features of ALK-negative ALCL and ALK-positive ALCL are nearly indistinguishable, with the only differentiation being the presence or absence of the ALK gene chromosomal rearrangements [11-13].

The ALK-negative ALCL primarily occurs in adults aged 50 years or older. This disease exhibits a slight male preponderance with a ratio of 1.5:1. This report presents a rare case of ALK-negative ALCL involving the lungs and mediastinum. Typically, this neoplasia primarily affects extranodal organs such as the skin, liver, lungs, and bones. The ALCL lung variant typically presents with rapidly progressing mediastinal lymphadenopathy and constitutional symptoms [14,15]. The patient's clinical presentation, including B-symptoms such as weight loss, night sweats, and lymph node involvement, was crucial in determining the diagnosis. As a result, the healthcare team ordered additional diagnostic studies, such as immunohistochemistry, to rule out poorly differentiated adenocarcinoma and explore the possibility of a lymphoid neoplasm [16].

The IPI is a clinical tool that aids in the risk stratification and prediction of survival rates for patients diagnosed with any of the peripheral T-cell lymphoma (PTCL) subtypes. It encompasses relevant prognostic factors such as the patient's age, general health status, LDH levels, disease stage, and extranodal involvement. Our patient had an intermediate IPI, which was determined by awarding one point for each of the following characteristics: age over 60 years, elevated double-hit lymphomas (DHL), and Eastern Cooperative Oncology Group (ECOG) performance status 2 (patient is capable of all self-care but cannot carry out any work activities and is up and about for more than 50% of waking hours). Patients with ALK-negative ALCL are typically diagnosed with an intermediate-high IPI, indicating a poor prognosis and a lower likelihood of responding well to treatment, as evidenced in this case [17,18].

**Figure 6:**
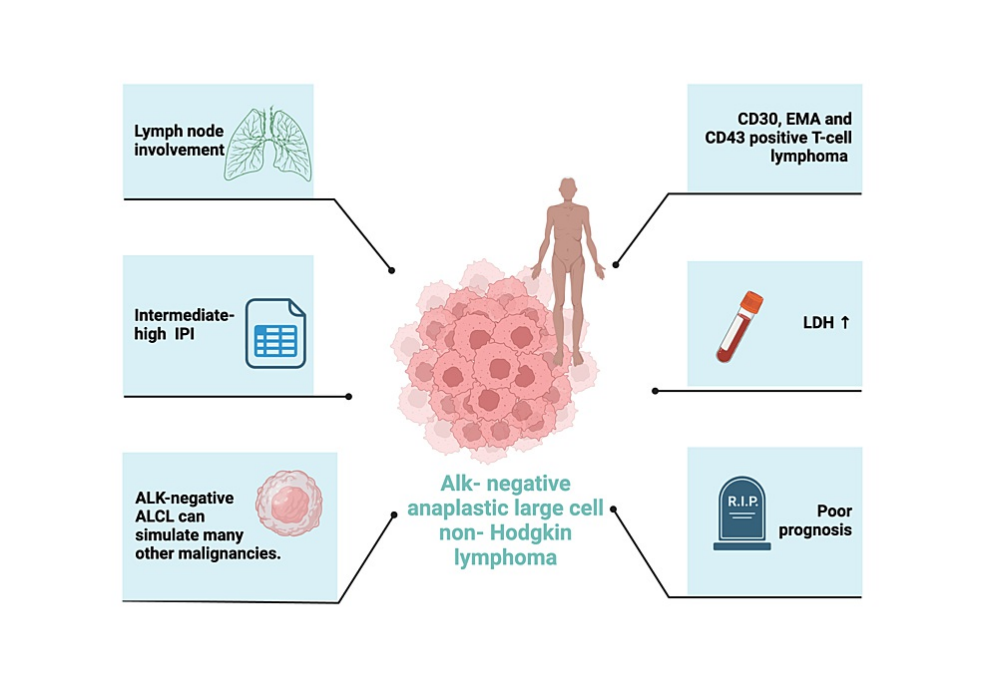
Characteristics of ALK-negative ALCL ALK: Anaplastic lymphoma kinase; ALCL: Anaplastic large cell lymphomas; IPI: International prognostic index; EMA: Epithelial membrane antigen; LDH: Lactate dehydrogenase. Image created with BioRender.com (Science Suite Inc., Toronto, ON, CAN).

According to research, the five-year overall survival rates of patients with ALK-positive ALCL are significantly higher than those with ALK-negative ALCL. Specifically, patients with ALK-positive ALCL have an overall survival rate of 79%, in contrast to the 38% rate observed in those with ALK-negative ALCL. Nonetheless, the unfortunate death of the patient in a short time serves as a reminder of the unfavorable prognosis associated with this neoplasia (Figure 6) [19].

The treatment for ALCL involves chemotherapy with cyclophosphamide, doxorubicin, vincristine, and prednisone (CHOP), which is commonly recommended as the initial approach for patients with ALK-positive ALCL. Conversely, although widely employed in ALK-negative ALCL patients, the CHOP regimen displays inferior outcomes with lower overall survival rates (<50%). Moreover, patients with a high-risk IPI classification treated with this first-line therapy exhibit survival rates of less than 30% [18].

Patients with relapsed or refractory ALCL, whose tumor cells strongly express CD30, can be treated with rescue chemotherapy using brentuximab vedotin (BV), while an autologous or allogeneic transplant of hematopoietic stem cells is available. Brentuximab vedotin is a monoclonal antibody against CD30 linked to the microtubule-disrupting cytotoxic compound monomethyl auristatin E. This regimen has shown better overall survival rates. It is a globally approved agent with good results in phase II trials of CD30-positive ALCL. And it is increasingly being included as an induction agent in combination with vedotin, cyclophosphamide, doxorubicin, and prednisone (BV+CHP). Therefore, it was decided to opt for this chemotherapy as a clear clinical benefit and better prognosis have been seen at standard doses in patients with ALCL [14,20-23].

The phase III ECHELON-2 study was conducted to compare the efficacy and safety of CHOP versus BV+CHP regimens in ALCL patients. This study included 316 patients, out of which 98 had ALK-positive disease and 218 had ALK-negative disease. The study revealed that patients who received BV+CHP combination therapy demonstrated better outcomes than those who received CHOP alone. Furthermore, both treatment groups experienced similar adverse effects. Despite these findings, there remains a significant research gap in the effectiveness of BV-based combinations, particularly in patients with ALK-negative ALCL [24]. Hence, there is a need for further research in this field to develop more effective therapies.

## Conclusions

The low incidence of this neoplasm and the rarity of the case due to the involvement of the lung and mediastinum mandate the exclusion of a vast array of differential diagnoses of mediastinal pathology, such as solid cancers and reactive processes. Despite the heterogeneity of its morphology and its atypical immunophenotype within lymphomas, the clinical presentation and histology of this neoplasia mirror those of other types of cancer. Thus, a comprehensive approach is imperative, utilizing suitable diagnostic tools and a well-executed histological study inclusive of immunohistochemistry to achieve a precise diagnosis and determine the optimal treatment plan, given the poor prognosis associated with this type of neoplasia.
